# A One-Layer Satellite Surface Energy Balance for Estimating Evapotranspiration Rates and Crop Water Stress Indexes

**DOI:** 10.3390/s90100001

**Published:** 2009-01-05

**Authors:** Salvatore Barbagallo, Simona Consoli, Alfonso Russo

**Affiliations:** Department of Agricultural Engineering, University of Catania / Via S. Sofia, 100 - 95123 Catania Italy; E-Mails: sbarbaga@unict.it; alfonso.russo@unict.it

**Keywords:** Evapotranspiration, satellite detection, surface energy balance, water stress indexes

## Abstract

Daily evapotranspiration fluxes over the semi-arid Catania Plain area (Eastern Sicily, Italy) were evaluated using remotely sensed data from Landsat Thematic Mapper TM5 images. A one-source parameterization of the surface sensible heat flux exchange using satellite surface temperature has been used. The transfer of sensible and latent heat is described by aerodynamic resistance and surface resistance. Required model inputs are brightness, temperature, fractional vegetation cover or leaf area index, albedo, crop height, roughness lengths, net radiation, air temperature, air humidity and wind speed. The aerodynamic resistance (r_ah_) is formulated on the basis of the Monin-Obukhov surface layer similarity theory and the surface resistance (r_s_) is evaluated from the energy balance equation. The instantaneous surface flux values were converted into evaporative fraction (EF) over the heterogeneous land surface to derive daily evapotranspiration values. Remote sensing-based assessments of crop water stress (CWSI) were also made in order to identify local irrigation requirements. Evapotranspiration data and crop coefficient values obtained from the approach were compared with: (i) data from the semi-empirical approach “K_c_ reflectance-based”, which integrates satellite data in the visible and NIR regions of the electromagnetic spectrum with ground-based measurements and (ii) surface energy flux measurements collected from a micrometeorological tower located in the experiment area. The expected variability associated with ET flux measurements suggests that the approach-derived surface fluxes were in acceptable agreement with the observations.

## 1. Introduction

During the past decades, considerable efforts have been made in the use of remote sensing to evaluate the interactions between land surface and atmospheric processes over a wide range of scales (spatial and temporal) [1-4]. Energy exchange at the land-atmosphere interface occurs through processes associated with surface radiation and energy balance. These processes are controlled by complex factors including surface resistance (which controls the partitioning of energy into heat and water vapour) surface roughness (which causes atmospheric turbulence near the surface, influencing the transfer rates of heat and water vapour into the atmosphere), amount and nature of vegetation cover, thermal soil properties and soil moisture content [5-7]. One of the appeals of remote sensing is that it facilitates evaluation of energy and water balances that can be used for monitoring crop water requirements, crop water stress and the effects of climate change within large areas or individual fields [8-10].

Generally, two main satellite-based approaches were applied over irrigated agricultural areas to estimate crop water needs in terms of evapotranspiration flux: (1) the reflectance-based crop coefficient method [11-12] and (2) the energy balance method [13-14]. In the reflectance-based crop coefficient method, spectral inputs in the red and near-infrared bands from ground-based radiometers, airborne sensors or satellite images are used to obtain vegetative indices (i.e. WDVI, NDVI, SAVI, etc.) related to the basal crop coefficient [15]. One of the main advantages of using crop coefficients is that they provide an underlying model for interpolation between satellite images over time. In the energy balance method, remotely sensed data in the thermal infrared spectrum are used to model different components of the energy balance equation, such as net radiation, soil heat flux, sensible heat flux and latent heat flux. The method is more complex to apply, requiring calibrated satellite imagery and the use of an atmospherically corrected thermal infrared band, which for most satellite instruments translates into lower spatial resolution [16].

Modelling evapotranspiration on a large scale with heterogeneous surface conditions requires a great deal of simplification, while preserving the key surface elements which control energy balance. For example, in the absence of vegetation, the surface characteristics can be described by surface albedo, emissivity, roughness length, and soil moisture content. When vegetation is present, the surface parameterization becomes more complex because vegetation transpiration is affected by the morphological and physiological characteristics of vegetation. It follows that when surface temperature is measured by a satellite (or an aircraft), the complex surface status can be lumped together, the remotely-sensed surface temperature representing a spatially integrated thermal status of the surface [10]. Based on these considerations, actual evapotranspiration from a heterogeneous surface can be conceptualized as a one-layer process from an average surface transferring sensible and latent heat [10, 17].

In this paper, a one-layer resistance (surface and aerodynamic) model was applied to estimate evapotranspiration fluxes over a semi-arid agricultural area in Eastern Sicily (Italy). Remotely sensed data of spatially integrated surface characteristics were combined with ground-based agro-meteorological measurements. Satellite data was provided by the Landsat Thematic Mapper TM5 sensor during June-September 2007. The objectives of the study were (i) to compare satellite-based energy balance surface fluxes with micrometeorological data from a flux tower that could be used to scale ET over orange orchards; (ii) to apply a reflectance-based approach to derive relationships between Landsat-based vegetation indices and crop coefficients (K_c_) and (iii) to recognize plant water stress by satellite-based estimates of the crop water stress index (CWSI).

## 2. Description of the modeling approach

### 2.1. The surface energy balance approach

**Figure 1.**
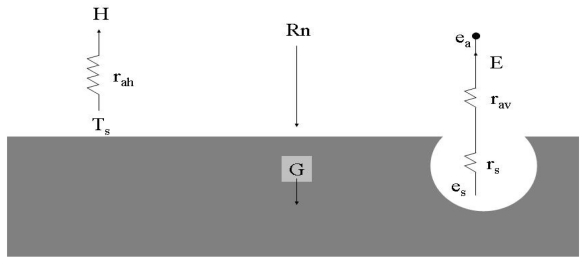
Schematic diagram of one-source thermal-based model for energy balance terms.

The complex relationships between surface temperature, vegetation features and energy flux have been analysed by several authors [10, 18-20] and numerous studies have proposed the use of one-dimensional (1-D) models to describe radiation conduction and turbulent transport mechanisms which influence energy balance and surface temperature [18] (Figure 1). Generally, all such models are based on energy conservation principles which dictate that net radiation R_N_ (W m^-2^) is balanced by the soil heat flux (G, W m^-2^), sensible heat flux (H, W m^-2^) and latent heat flux (LE, W m^-2^) at the surface:

(1) $${\text{R}}_{\text{N}}=\text{G}+\text{H}+\text{LE}$$

Generally, it is assumed that R_N_ may be easily computed, and G is parameterized in a straightforward fashion (as a simple proportion of R_N_). The two remaining terms, H and LE, are turbulent flux quantities and are the most difficult to estimate.

In the study, net radiation was estimated as:

(2) $${\text{R}}_{\text{N}}={\text{R}}_{\text{s}}\left(1-\text{r}\right)+{ɛ}_{\text{a}}\sigma {\text{T}}_{\text{a}}^{4}-{ɛ}_{\text{s}}\sigma {\text{T}}_{\text{s}}^{4}$$

where R_s_ is the incoming short wave radiation (Wm^-2^) measured by pyranometers, σ is the Stefan-Boltzman constant (5.67 10^-8^ Wsm^-2^K^-4^), ε is emissivity and T is the temperature (K) with the subscripts ‘a’ and ‘s’ for air and surface respectively; the surface albedo (r) is computed from the formulation proposed by Menenti in 1984 (see Table 1).

Soil heat flux was calculated by assuming that the ratio G/R_N_ is related to the fractional vegetation cover [8]. For vegetated surfaces the term G/R_N_ is less with respect to bare soil because of the partial extinction of net radiation by the vegetation cover. Because spectral vegetation indices (VI_s_) are proportional to the net radiation extinction by the canopy, the VI can be used as a linear scaling factor to estimate G/R_N_ over vegetated fields [25]. In order to avoid the calibration of the relationship between G/R_N_ and VI_s_, it is assumed here that G/R_N_ is related to the fractional vegetation cover by Eq. 3. The fractional vegetation cover is estimated from LAI.

(3) $$\left(\text{G}/{\text{R}}_{\text{N}}\right)={\text{f}}_{\text{v}}{\left(\text{G}/{\text{R}}_{\text{N}}\right)}_{\text{veg}}+\left(1-{\text{f}}_{\text{v}}\right)\cdot {\left(\text{G}/{\text{R}}_{\text{N}}\right)}_{\text{soil}}$$

with (G/R_N_)_veg_=0.05, (G/R_N_)_soil_=0.315, and f_v_ estimated from LAI.

The terms of Eq. (1) are modelled using a 1-D flux-gradient expression based on a convection analogue to Ohm's law:

(4) $$\text{H}=\rho {\text{C}}_{\text{p}}\frac{{\text{T}}_{\text{s}}-{\text{T}}_{\text{a}}}{{\text{r}}_{\text{ah}}}$$

where ρ is air density (Kg m^-3^), C_p_ is the specific heat of air at a constant pressure (J kg^-1^ K^-1^) and r_ah_ is the aerodynamic resistance for sensible heat (s m^-1^). Eq. 4 is a one-layer bulk transfer equation based on the assumption that the radiometric temperature measured by a thermal infrared radiometer is identical to aerodynamic temperature. In fact, in the case of full canopy cover, there is near-equivalence between these two temperatures and it is found that estimates of evapotraspiration using radiometric temperatures are in good agreement with observed values [10, 26-27].

Surface temperature (T_s_) is the thermal emission from the landscape surface, including vegetated surfaces, as well as other surfaces (such as bare soil). In the study T_s_ was derived from band 6 TIR of Landsat TM5 using the model developed by Sobrino *et al.* in 2004:

(5) $${\text{T}}_{\text{s}}=\frac{{\text{T}}_{\text{B}}}{1+\left(\lambda \cdot \overset{{\text{T}}_{\text{B}}}{\;}/\underset{\text{r}}{\;}\right)\ln(ɛ)}$$

where λ is the wavelength of emitted radiance (λ=11.5), r=h·c·σ equalling 1.438 10^-2^ mK, where h is Planck's constant (6.626 10^-34^ J s), c the velocity of light (2.998 10^8^ m s^-1^) and σ the Boltzman constant (1.38 10^-23^ JK^-1^); emissivity ε was estimated through [28]:

(6) $$ɛ={\text{f}}_{\text{v}}{ɛ}_{\text{v}}+\left(1-{\text{f}}_{\text{v}}\right)\cdot {ɛ}_{\text{s}}$$

where ε_v_ and ε_s_ denote emissivity of vegetation (0.985) and soil (0.960). The fractional vegetation cover f_v_ is related to leaf area index (LAI), f_v_ = 1 − e^−0.5·LAI^ [9]. By applying the inverse of Plank's radiation equation, spectral radiance in the thermal band was converted to brightness temperature T_B_:

(7) $${\text{T}}_{\text{B}}=\frac{{\text{K}}_{2}}{\ln\left(\frac{{\text{K}}_{1}}{{\text{L}}_{\lambda }+1}\right)}$$

where K_1_ and K_2_ are calibration constants (equal to 607.76 W m^-2^ sr^-1^ μm^-1^ and 1260.56 K respectively) defined for Landsat 5 TM sensor [29]; L_λ_ is the pixel value as radiance (W m^-2^ sr^-1^ μm^-1^), L_λ_=G·(CV_DN_)+B, with CV_DN_ the pixel value as digital number, G and B the gain and the offset for TM6, respectively [30]. The inverse of Planck's law, used to derive T_s_, can be interpreted as a correction of the atmospheric and emissivity effects on the data measured by the sensor [31].

Latent heat transfer is expressed as:

(8) $$\text{LE}=\frac{\rho {\text{C}}_{\text{p}}}{\gamma }\frac{{\text{e}}_{\text{s}}({\text{T}}_{\text{s}})-{\text{e}}_{\text{a}}}{{\text{r}}_{\text{av}}+{\text{r}}_{\text{s}}}$$

where γ is the psychometric constant (0.066 kPa C^-1^), e_s_(T_s_) is the saturated vapour pressure at the surface temperature (kPa), e_a_ is the vapour pressure at the reference height (kPa), r_av_ is the physiological resistance (s m^-1^) to moisture transport at the surface. The surface resistance r_s_ (s m^-1^) to vapour transfer exerts strong control on the partitioning of available energy (R_N_-G) between H and LE.

The aerodynamic resistance r_ah_ of eq. 4 was calculated on the basis of the Monin-Obukhov surface layer similarity theory [32]:

(9) $${\text{r}}_{\text{ah}}=\frac{\left[\ln\left(\frac{\text{z}-\text{d}}{{\text{z}}_{\text{oh}}}\right)-\Psi_{\text{sh}}\right]\times \left[\ln\left(\frac{\text{z}-\text{d}}{{\text{z}}_{\text{om}}}\right)-\Psi_{\text{sm}}\right]}{{\text{k}}^{2}\cdot \text{u}}$$

where z_oh_ e z_om_ are roughness lengths for sensible heat and for momentum (m), respectively; z_om_=0.13·h_c_ (with h_c_ the mean height of the crop in meters); z_oh_=0.1·z_om_ [26]; d=0.66·h_c_ is the zero-plane displacement height (m); Ψ_sh_ e Ψ_sm_ are the stability correction functions for momentum and sensible heat; k (0.4) is von Karman's constant; u (m s^-1^) is the wind speed at level z (10 meters). The stability correction functions were determined with the Businger-Dyer formulations [33] for unstable conditions [34]:

(10) $$\Psi_{\text{sm}}=2\ln\left[\frac{1+\text{x}}{2}\right]+\ln\left[\frac{1+{\text{x}}^{2}}{2}\right]-2\arctan(\text{x})+\pi /2$$

(11) $$\Psi_{\text{sh}}=2\ln\left[\frac{1+{\text{x}}^{2}}{2}\right]$$

where x=(1-16R_i_)^1/4^, with ${\text{R}}_{\text{i}}=\frac{\text{g}\left({\text{T}}_{\text{s}}-{\text{T}}_{\text{a}}\right)\cdot \left(\text{z}-\text{d}\right)}{{\text{T}}_{\text{a}}\cdot {\text{u}}^{2}}$ the Richardson number, and g is the acceleration due to gravity (m s^-2^).

Surface resistance is determined by substituting eqs. (4) and (8) into eq. (1), without making a distinction between soil evaporation and plant transpiration:

(12) $$r_{s}=\frac{\left(e_{s}\left(T_{s}\right)-e_{a}\right)}{\gamma \left[\left(R_{N}-G\right)/\rho C_{p}-\left(T_{s}-T_{a}\right)/r_{ah}\right]}-r_{av}$$

in which the physiological resistance r_av_ was considered equal to r_ah_ [10].

The applied method calculates instantaneous evapotranspiration (LE) estimates only. The extrapolation of LE into daily estimates, which most interests agricultural water management, was based on evaporative fraction (EF) [14]:

(13) $$\text{EF}=\frac{\text{LE}}{{\text{R}}_{\text{N}}-\text{G}}$$

Daily evapotranspiration ET_24_ (mm d^-1^) values were then calculated by the following equation:

(14) $$ET_{24}=EF\frac{R_{N,24}}{L}$$

where L (MJ m^-2^ mm^-1^) is the latent heat of vaporization and R_N,24_ is the daytime (09:00 to 16:00 LST) net radiation measured by a micrometeorological flux tower.

### 2.2. The crop water stress index

In the study, the analysis of the crop water stress index (CWSI) [35-36] was used to indicate plant water stress as measure of the transpiration rate occurring from the vegetated surface (using canopy temperature data). CWSI values of zero indicate no water stress, and values of 1 represent maximum water stress. The CWSI was computed as [35]:

(15) $$\text{CWSI}=\frac{\left({\text{T}}_{\text{s}}-{\text{T}}_{\text{a}}\right)-{\left({\text{T}}_{\text{s}}-{\text{T}}_{\text{a}}\right)}_{\text{lower}}}{{\left({\text{T}}_{\text{s}}-{\text{T}}_{\text{a}}\right)}_{\text{upper}}-{\left({\text{T}}_{\text{s}}-{\text{T}}_{\text{a}}\right)}_{\text{lower}}}$$

where (T_s_-T_a_) is the measurement, (T_s_-T_a_)_lower_ is the theoretical minimum value for (T_s_-T_a_) and (T_s_-T_a_)_upper_ is the theoretical maximum value for (T_s_-T_a_).

Jackson *et al.*, using a steady state energy balance of a crop canopy, developed a theoretical CSWI where:

(16) $${\text{T}}_{\text{s}}-{\text{T}}_{\text{a}}=\frac{{\text{r}}_{\text{ah}}\left({\text{R}}_{\text{N}}-\text{G}\right)}{\rho {\text{C}}_{\text{p}}}\times \frac{\gamma \left(1+{\text{r}}_{\text{s}}/{\text{r}}_{\text{ah}}\right)}{\Delta +\gamma \left(1+{\text{r}}_{\text{s}}/{\text{r}}_{\text{ah}}\right)}-\frac{\text{VPD}}{\Delta +\gamma \left(1+{\text{r}}_{\text{s}}/{\text{r}}_{\text{ah}}\right)}$$

in which VPD is the vapor pressure deficit (kPa); the other variables of Eq. 16 are satellite-based estimates and were introduced in the previous paragraph.

Eq. 16 is used to calculate the theoretical minimum and maximum values of (T_s_-T_a_), using inputs of R_N_, G, r_ah_, r_s_, and VPD, with T_s_ the pixel composite temperature of vegetation and soil. The maximum theoretical value for (T_s_-T_a_) was evaluated assuming r_s_ approaches infinity:

(17) $${\left({\text{T}}_{\text{s}}-{\text{T}}_{\text{a}}\right)}_{\text{upper}}=\frac{{\text{r}}_{\text{ah}}\left({\text{R}}_{\text{N}}-\text{G}\right)}{\rho {\text{C}}_{\text{p}}}$$

The minimum theoretical value for (T_s_-T_a_) was defined by setting r_s_ equal to zero in Eq. 16:

(18) $${\left({\text{T}}_{\text{s}}-{\text{T}}_{\text{a}}\right)}_{\text{lower}}=\frac{{\text{r}}_{\text{ah}}\left({\text{R}}_{\text{N}}-\text{G}\right)}{\rho {\text{C}}_{\text{p}}}\times \frac{\gamma }{\Delta +\gamma }-\frac{\text{VPD}}{\Delta +\gamma }$$

### 2.3. The K_c_ reflectance-based approach

The reflectance-based crop coefficient method [12] consists of the direct application of a theoretical ET equation to define K_c_ [12, 37]:

(19) $${\text{K}}_{\text{c}}=\frac{{\text{ET}}_{\text{c}}}{{\text{ET}}_{0}}$$

While reference evapotranspiration (ET_0_) accounts for variations in weather and offers a measure of the ‘evaporative’ demand of the atmosphere, crop coefficients (K_c_) account for the difference between reference (ET_0_) and potential (ET_c_) crop evapotranspiration. The main factors affecting that difference are light absorption by the canopy, canopy roughness (which affects turbulence), crop physiology, leaf density and surface wetness. Crop coefficient values (K_c_) thus estimated were expressed as follows [55]:

(20) $$\begin{array}{cccc} K_{c}=\sum_{i=0}^{4}C_{i}LAI^{i} & \text{with} & C_{i}=a_{i}+b_{i}r & i=0,.1,2,.3,.4 \end{array}$$

where the coefficients *a* and *b* of the polynomial equation were determined as functions of climatic data (net radiation R_N_, air temperature T, air humidity RH, and wind speed u) measured by the automatic stations located within the study-area and canopy properties (*LAI*, *albedo* r) were determined using remote sensed data [12, 38].

Eq. 20 evidences that the values of K_c_ is not only dependent on the canopy variables, but also on the meteorological data. Canopy variables, except albedo which depends also on the soil surface moisture, change slowly over time. In the calculation of K_c_ by means of Eq. 20, albedo is linearly interpolated between two consecutive satellite passes. As a consequence, the variation of r with changing surface soil moisture is not considered. This assumption may appear rather questionable however, especially for surface with partial ground cover. The influence of soil moisture on the spectral properties in such conditions was analysed by Kustas et al. (1994) [39]. In this case, r was measured by means of low-altitude spectral data on different dates. By comparing observation before and after several rainfall events at eight sites, the maximum observed variation of r was 0.03. The effect of variation of this order of magnitude on K_c_ is negligible.

By multiplying pixel-wise, the crop coefficient of Eq.20 and the ET_0_ values, the potential evapotranspiration (ET_c_) fluxes of the crop were estimated. This procedure avoids the need for frequent acquisition of satellite data, since they are used to determine *albedo* and LAI which don't change very rapidly.

## 3. Application of the proposed approach

### 3.1. Experimental site and micrometeorological energy fluxes

**Figure 2.**
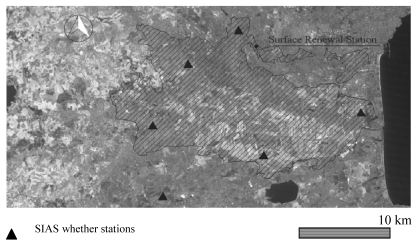
Catania Plain irrigation area.

The Catania Plain area is the largest agricultural district in Sicily (Italy), with an area of about 50,000 ha (Table 2 and Figure 2). It is characterized by citrus orchards for more than 90% of the irrigated area (about 18,000 ha), the other cultivated crops being fodder, artichokes, watermelons and vegetables. The irrigation water for the Plain is provided by the Salso-Simeto river system. Irrigation applications are delivered from collective water distribution networks at fixed intervals (generally 21 days during the irrigation season) and are applied at farm level by micro-sprayers. The climate is semi-arid and the annual potential ET exceeds by about 30% the mean annual rainfall (about 500 mm) [40].

During June-September 2007, surface energy fluxes, meteorological data and radiometric temperatures were measured by a micrometeorological flux tower located in an experimental area with a fetch of more than 200 m in all directions. Generally, fetch represents the distance from the micrometeorological tower in which the canopy characteristics (crop type and crop height) are uniform.

The variation of the main components of the energy balance equation (eq.1) was computed at 1 hour intervals throughout the monitored period. Net radiation R_N_ was measured using a “Kippen & Zonen” net radiometer mounted at about 1 m above the orchard canopy (crop mean height of about 3.5 m). Soil heat flux density G was measured using three REBS/HFP01 soil heat flux plates and two Campbell Scientific, Inc. ‘CS’ ® TCAV soil averaging temperature sensors to account for soil heat storage above each heat flux plate.

The plates were inserted horizontally into the soil at a depth of 0.05 m, and the soil thermocouples were placed 0.01-0.04 deep. The plates and temperature sensors were placed in and outside of the tree rows to obtain a good estimate of soil heat flux within the orchard. In particular, the control points were placed near the tree (shaded point), at 1/4 of the distance between orchard row (penumbral effect), and at 1/2 of the distance between rows (illuminated point). The volumetric heat capacity of soil was used to compute changes in heat storage above the flux plates. It was computed according to:

(21) $$\text{Cv}=(0.837\rho_{\text{b}}+4.190\theta )10^{6}$$

where ρ_b_ is the bulk density and θ is the volumetric water content measured by three TDR CS616 located at the same depth of the heat flux plates. High frequency temperature data was collected at 4 Hz using two 76.2 μm diameter fine-wire thermocouples mounted at 0.5 meters above the canopy top. When plotted against time the temperature traces show ramp-like characteristics, which are used to estimate heat fluxes using a conservation of energy equation [41-44]. The temperature data was analyzed to determine the mean ramp amplitude (a) and the inverse ramp frequency (d+s) using a structure function [45] and time lags of 0.25 and 0.50 seconds for each of the two thermocouples. Sensible heat flux was calculated as:

(22) $$\text{H}=\alpha \rho {\text{C}}_{\text{p}}\left(\frac{\text{a}}{\text{d}+\text{s}}\right)\text{z}$$

Factor α is a correction term for unequal heating below the sensors that depends on the measurement height (z), on canopy structure and thermocouple size. In combination, half-hourly data on H, R_N_ and G were used to calculate latent heat flux density (LE) as the residual of the energy balance equation. The actual crop ET (ET_a_) was computed by dividing hourly the means of LE by the latent heat of vaporization L=2.45 MJ m^-2^ mm^-1^.

Generally, crop coefficients are determined by calculating the ratio K_c_ = ET_c_/ET_0_, where ET_c_ is the evapotranspiration of a well-watered crop. Since these orchards are well managed, it is assumed that there was little or no transpiration reducing water stress and ET_a_ ≈ ET_c_.

The spatial distribution of solar radiation, air temperature, vapour pressure, relative humidity, wind speed and direction, and rainfall came from six automatic weather stations (Campbell Scientific, Logan, UT) located in the Catania Plain area (see Figure 2). These weather stations are part of the Sicilian Agrometeorological Information Service (SIAS). Hourly weather data was used to calculate reference evapotranspiration ET_0_ (eq. 23) using the FAO 56 Penamn-Monteith equation for short crops [37, 46]. Hourly ET_0_ values were summed over 24-hour periods to obtain daily ET_0_ data:

(23) $$ET_{0}=\frac{0.408\Delta \left(R_{N}-G\right)+\gamma \frac{900}{T+273}u_{2}\left(e_{s}-e_{a}\right)}{\Delta +\gamma \left(1+0.34u_{2}\right)}$$

In Eq. 23, Δ is the slope of saturation vapour pressure at air temperature (kPa °C^-1^), γ is the psychrometric constant (kPa °C^-1^), T is the daily mean air temperature (°C), u_2_ is the mean wind speed in m s^-1^ and e_s_-e_a_ is the vapour pressure deficit (kPa).

Soil moisture was monitored continuously using the Time Domain Reflectrometry (TDR) technique in different fields within the experimental area, at soil depths of 15, 30 and 60 cm. The soil type in the experimental field was clay [47] with a soil moisture content at field capacity of about 35% and an available water holding capacity of about 190 mm m^-1^ on an oven dry weight loss basis.

Leaf area index (LAI) values were measured with a Licor LAI-2000 digital analyzer at regular intervals during the satellite acquisitions.

### 3.2. Processing satellite-based data

The satellite data consisted of Landsat Thematic Mapper TM5 images (Table 3) acquired on June 14^th^, July 22^nd^, August 17^th^ and September 8^th^ 2007. The images were geometrically rectified to a Universal Transversal Mercator projection system (UTM) by using a linear transformation of coordinates and the nearest-neighbour resampling method for pixel reflectance values [48]. The reflectance values in the VIS/NIR region of the electromagnetic spectrum were calculated from the images, or at the top of atmosphere or by applying a correction for the atmospheric effects. In the first case, the reflectance at the top of atmosphere (*ρ_TOA,λ_*) was computed according to the following equation:

(24) $$\rho_{\text{TOA},\lambda }=\frac{\pi \cdot {\text{L}}_{\lambda }\cdot {\text{d}}^{2}}{{\text{E}}_{0}^{\lambda }\cdot \cos\theta }$$

where *L_λ_* is the spectral radiance at the sensors (mW/cm^2^·sr·μm); *d* is the Earth-Sun distance in Astronomical Units; *E^0^_λ_* is the extraterrestrial solar irradiance (W/m^2^); *θ* is the solar zenith angle in degrees.

In the second case, atmospherically corrected reflectance values were derived by means of the ATCOR procedure [49] assuming constant atmospheric conditions over the image and different types of standard atmospheric profiles, i.e. mid-latitude summer, rural, maritime, etc. The correction, based on the mid-latitude summer profile, was considered the most reliable comparing the resulting spectra for some targets i.e. vegetation, water and soil. Thermal band 6 needs no calibration, since the derived surface temperature data accords well with the surface temperature data from the infrared thermometers (CS Model IRTS-P) mounted at a height of 4 m above ground and pointing 45° towards the surface. The average range of processed Landsat TM temperatures is 29-44°C, and the mean air temperature (measured at the six weather stations at about 10 meters of height) is between 30-43°C at the local time (10:00 a.m.) of satellite overpass. Landsat TM pixels encompassing the tower site were used to establish relationships between flux tower ET and the satellite data for energy flux and vegetation indices.

## 4. Results and Discussion

### 4.1. Comparing the model estimates of energy flux with micrometeorological measurements

**Figure 3.**
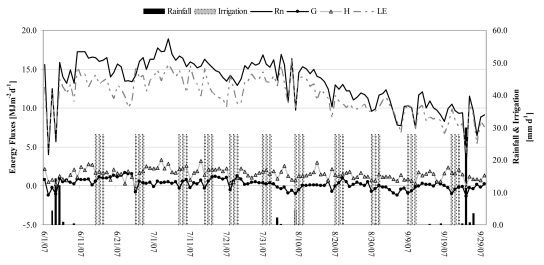
Daily values of energy flux, rainfall and irrigation rates during June-September 2007.

The results that follow are based on the models described in the previous sections using satellite and field data collected during the period of experiment in 2007. The micrometeorological data recorded by the flux tower in the orchard is used to illustrate the suitability of the one-layer approach for computing evapotranspiration rates. Figure 3 shows the tower-based daily energy balance calculations from June - September 2007. Sensible heat flux (H) was between zero and 3.4 MJ m^-2^ d^-1^ with an average of 2.5 MJ m^-2^ d^-1^. Latent heat flux (LE) averaged 11.6 MJ m^-2^d^-1^, varying between 4.2 and 16.2 MJ m^-2^d^-1^. Net radiation (R_N_) values varied between a maximum of 18.9 and a minimum of 2.7 MJ m^-2^ d^-1^, with an average of 13.3 MJ m^-2^d^-1^. The lower R_N_ values were most likely caused by precipitation and the greater *albedo* due to cloud cover. An average solar radiation (R_s_) near 19.5 MJ m^-2^d^-1^ was recorded during the monitoring. On a daily basis the G term was generally close to zero.

**Figure 4.**
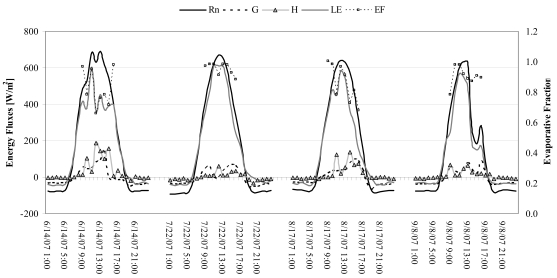
Hourly energy flux and EF at the micrometeorological station in the study area.

Micrometerological tower fluxes during the satellite overpass (10:00 a.m. local time) are plotted in Figure 4. In general, agreement between the modeled and observed fluxes was good. The observed mean energy fluxes were respectively 521.5, 42.5, 31.7 and 447.3 W m^-2^ for net radiation (R_N_), soil heat flux (G), sensible (H) and latent heat (LE) flux densities. The energy fluxes obtained by processing TM bands during the satellite acquisition dates had a relatively narrow spatial distribution (maximum time variation of about 24%) at the tower site, with average values of 570, 40.4, 45.6 and 408.3 W m^-2^ respectively for net radiation (R_N_), soil heat flux (G), sensible (H) and latent heat (LE) flux densities. The daytime (from 09:00 to 16:00 LST) averages and standard deviation of evaporative fraction (EF) during the satellite acquisitions (see Fig. 4) were computed in order to justify the assumption of a constant EF in Eq. 14. In particular, the mean (about 0.90) of daytime EF, which characterizes the partition of the energy budget at the daily time scale, varied little (0.06) based on average cloudiness. The temporal variability of the partitioning, expressed in terms of EF daily standard deviation, reached a maximum of 14%. The experiment showed that the evaporative fraction computed from flux measurements at 4 hours past sunrise tends to increase very slowly, thus to assume that the underestimation in daytime average would be not significant.

**Figure 5.**
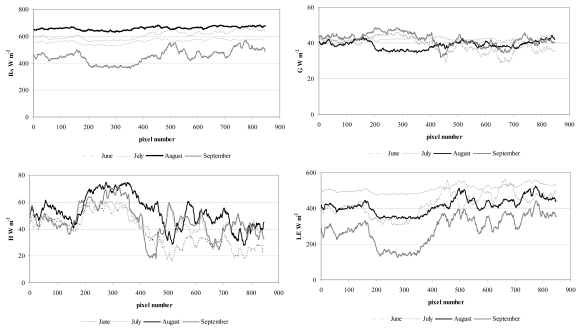
Spatial distribution of remote sensed energy flux (satellite acquisition from June to September 2007).

The spatial variability of surface energy fluxes from Landsat scenes of about 850 mixed pixels was depicted in Figure 5. The study revealed that the amount of energy available for physical and biological processes over the crop (R_N_) varied from a maximum of 638 W m^-2^ on August 17^th^ 2007 to a minimum of 361 W m^-2^ on September 8^th^. The main variation of latent heat flux density (LE) occurred due to variations of solar radiation, temperature, leaf area index and soil moisture.

The LE variation was from 564 W m^-2^ on July 22^nd^ to 127 W m^-2^ on September 8^th^. The soil heat flux range was 28.8 - 48.5 W m^-2^, with a maximum spatial variation of 10%. Sensible heat flux from the surface to the atmosphere (H) varied from 74.7 W m^-2^ on August 17^th^ to 17.5 on September 8^th^ 2007, with a mean of 45.6 W m^-2^ and spatial variation of 24%. Daily satellite ET_24_ (mm d^-1^) values strongly (R^2^=0.8, with R^2^ the determination coefficient) correlated with NDVI and LAI. ET correlated more weakly (R^2^=0.37) with net radiation (R_N_) across the period, showing that the plants were not radiation-limited most of the time. Hence, ET was mainly determined by the amount of green vegetation or functioning vegetation in the agricultural field which is typical for semi-arid landscapes [50].

**Figure 6.**
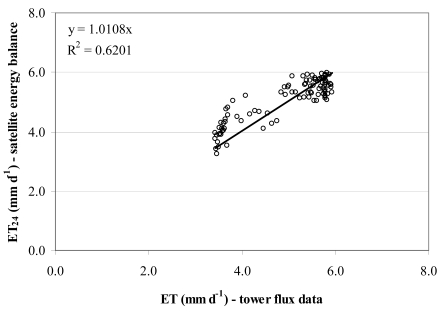
Comparison of ET_24_ values predicted from the satellite energy balance approach with those calculated from tower flux measurements (data were paired from June 14^th^ to September 8^th^ 2007).

Because of the limited frequency of Landsat images, daily evapotranspiration (ET_24_) data was estimated by linearly interpolating the variable values for the periods in between two consecutive images, in the same spatial resolution as the original satellite scenes. The calculated ET_24_ values compare fairly well to the tower flux estimates of evapotranspiration using the Surface Renewal technique (Figure 6). Mean ET_24_ values across June-September 2007 were 4.98 (5.30) and 5.08 mm d^-1^, respectively, from a satellite energy balance approach and from tower flux measurements with a temporal variability of about 15%.

**Figure 7.**
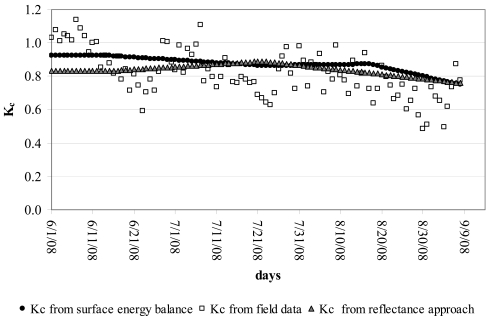
Kc from field data compared with Kc from the satellite approach.

In Figure 7, the satellite-based crop coefficients (K_c_) computed as the ratio between ET_24_ (mm d^-1^) and the grass reference ET from eq. 23, were compared with tower flux crop coefficients and the results of the reflectance-based approach. Crop coefficients during June-September 2007 were in the ranges 0.75-0.92, 0.76-0.89 and 0.5-1.14 from respectively, satellite energy balance, reflectance-based approach and tower flux data. Maximum variability occurred with K_c_ tower flux data whereas the satellite-based K_c_ estimates were more uniform. On average (about 0.8), crop coefficients were slightly higher than those reported in the widely used FAO 56 [37] and FAO 24 [51] publications for orchards with about 70% ground cover, corrected for local humidity and temperature as suggested by Allen et al. in 1998. The higher K_c_ values from *in situ* measurements might be due to better available moisture for trees resulting from rainfall events (especially during the first week of June) and frequent irrigation with microsprayers. Linear correlations express the increase in K_c_ from the reflectance-based approach with NDVI. The linear trend presents a determination coefficients (R^2^) higher than 0.90, with minimal scatter around the regression lines. It must be inferred that the relationship K_c_-NDVI is strictly related to the selected crop and the specific conditions of that area. Furthermore, it is well suited to orchard crops with pretty steady LAI values.

**Figure 8.**
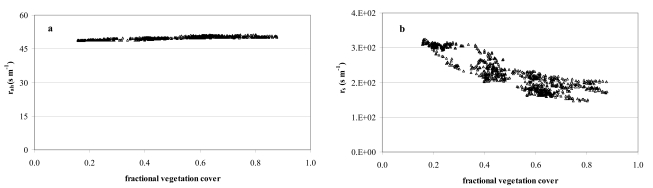
(a) Satellite-based aerodynamic resistance (r_ah_) as a function of f_v_; (b) Satellite-based surface resistance (r_s_) as a function of f_v_. A mean of 850 pixels was used to produce the graphs.

Figure 8 depicts the aerodynamic r_ah_ and surface r_s_ resistances as functions of the fractional vegetation cover (f_v_). Generally, a rather small range of r_ah_ values represents each f_v_ (Fig. 8a) part of which could be due to the quite homogeneous vegetation coverage (Table 4) (citrus orchards cover more than 90% of the site) and quite low spatial resolution of the surface temperatures T_s_ (120 m). Generally, the r_ah_ values were about 15% lower than those from other studies for citrus orchards [7]. Physically, we would expect that surface/plant systems with less resistance to energy flux transport would have less sensible heat and greater evaporation. The resulting value of roughness length is about an order of magnitude less than the height of the roughness elements, and in general agreement with that obtained for natural surfaces by Mahrt and Ek in 1993 [52] using aircraft measurements. In Figure 8b, r_s_ tends to change logarithmically with vegetation density variation. Dense vegetation (f_v_=0.88; r_s_ 145-160 s m^-1^) has been found for stressed canopies in semi-arid areas [53]. High surface resistances reflect dry soil surfaces and, generally, correspond to low soil moisture content at the irrigated site. This was confirmed by soil water content (TDR probes) at selected control sites reaching minimums of 27% when the T_s_-T_a_ difference was maximum. As expected, both T_s_-T_a_ and r_s_ are lower when LAI (and f_v_) is high.

**Figure 9.**
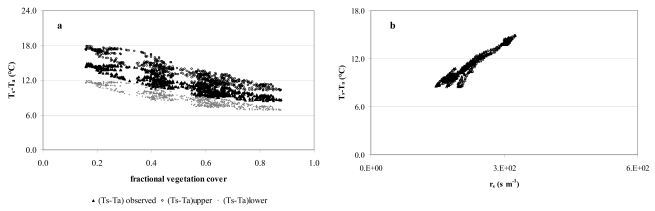
(a) Difference between T_s_ and T_a_ as a function of fractional vegetation cover; (b) Difference between T_s_ and T_a_ as a function of surface resistance r_s_. A mean of 850 pixels was used to produce the graphs.

In order to examine satellite observations for plant water stress, the theoretical upper and lower limits for T_s_-T_a_ are plotted against f_v_, together with the T_s_-T_a_ observations in Fig. 9a. It may be seen that the T_s_-T_a_ range is fairly small for a given f_v_ which represents homogeneous surface conditions. Generally, observed T_s_-T_a_ exceeded the theoretical lower limit, symbolizing the increase of surface control on LE probably caused by a reduction in the soil water availability and increased plant water stress (Fig. 9b). In the case study, (T_s_-T_a_)_lower_ and (T_s_-T_a_)_upper_ resemble the well-defined borderlines of the (T_s_-T_a_)-f_v_ scatter plot found in the literature [4, 54].

**Figure 10.**
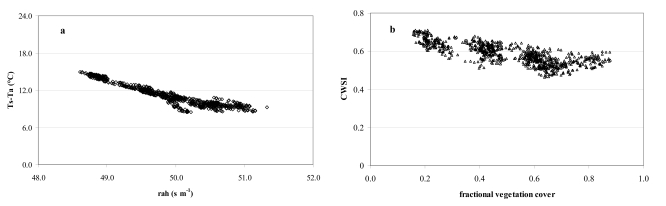
(a) Actual observation of T_s_-T_a_ in relation to the aerodynamic resistance; (b) CWSI compared to fractional vegetation cover.

Figure 10a shows the strong relationship between the actual T_s_-T_a_ observations plotted against r_ah_. It also illustrates that atmospheric turbulence is pretty steady and atmospheric resistance averages 48.6 s m^-1^ which may affect the atmospheric coupling between surface and atmosphere which causes differences between T_s_ and T_a_ [8]. A certain reduction in T_s_-T_a_ occurs when r_ah_ increases because of vertical air mixing.

Figure 10b reports the calculated values of CWSI versus fractional vegetation cover. The study revealed a mean CWSI from satellite data of 0.6 with a low variation (9%) for each value of f_v_. Energy flux data from the micrometeorological tower was also used to calculate the mean CWSI of 0.67 during satellite acquisitions. Previous studies [35-36, 55-56] on CWSI for many crops in different parts of the world highlighted that CWSIs higher than 0.6 indicate soil moisture depletion requiring irrigation.

**Figure 11.**
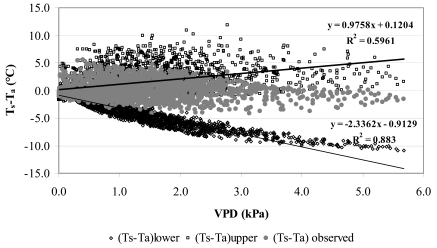
Relationship between T_s_-T_a_ and VPD of the orange orchard at the experimental site.

Using the tower-based surface energy flux data, Figure 11 depicts the corresponding canopy-air temperature differential as a function of VPD for well-watered (T_s_-T_a_)_lower_ and stressed plants (T_s_-T_a_)_upper_ which resulted from eqs. 17 and 18. The lower baseline shows an average decrease in canopy temperature of about 3°C for each 1kPa increase in VPD.

## 4. Conclusions

Determining evapotranspiration rates by remote sensing can help identify numerous factors such as droughts, sub-optimal irrigation and plant physiologies that are difficult to evaluate otherwise. Large-scale crop water monitoring requires remote sensing systems such as Landsat which have high resolutions and short return times (16 days but only if the atmosphere is free of clouds). In this study, a one-layer resistance model was used for the spatial estimation of evapotranspiration rates, vegetation indices and features using Landsat TM and local agro-meteorological data. The model formulates the transfer of sensible and latent heat fluxes between the surface and atmosphere using the concept of aerodynamic resistance and surface resistance. In the proposed approach, the assumption of near-equivalence between radiometric temperature measured by the thermal infrared radiometer and aerodynamic temperature was confirmed by the high values of fractional vegetation cover (mean of about 0.70) and percentage of ground cover by vegetation. Maps of atmospheric resistance, surface resistance, surface energy flux, evapotranspiration rates and CWSI were produced. The satellite-based estimates of ET rates compare well with the Surface Renewal data of evapotranspiration flux recorded at field level. However, the method should be tested thoroughly using an extended spatially distributed dataset. Crop coefficient values K_c_ as computed by the satellite reflectance-based approach had about the same range of variation of data on K_c_ derived by the one-layer energy balance method, with a mean of 0.8, slightly higher than the widely used FAO 56 data. The satellite-based estimate of surface resistance r_s_ tended to be lowest for dense vegetation (f_v_≅0.88) and highest for bare soil or canopies with intermediate vegetation cover. The surface resistance approaches 145-160 s m^-1^ for dense vegetation highlighting water stressed canopy conditions. A tendency to quite steady atmospheric resistance is partially due to the effect of fully vegetated pixels and the low spatial resolution of surface temperature T_s_.

The results of the satellite surface energy balance were further used to compute the upper and lower theoretical limits of T_s_-T_a_ for each image's pixels. In particular, the dependency of T_s_-T_a_ lower and upper limits on the fractional vegetation cover and surface resistance was demonstrated. Derived and measured CWSIs were in good accordance and had a mean of about 0.6 which indicates a certain soil moisture depletion.

Finally, estimation of ET within wide spatial scales by one-layer models and integration of ground-based meteorological data with satellite observations is a useful tool for quantifying and controlling water consumption especially in areas of limited water supply.

## Figures and Tables

**Table 1.** Vegetation parameters and vegetation indices analysed in the work.

Indicators*	**Expression**	**Parameters**	**Reference**
Normalized Difference Vegetation Index	$\text{NDVI}=\frac{\rho_{i}-\rho_{r}}{\rho_{i}+\rho_{r}}$		[21]
Weighted Difference Vegetation Index	$\text{WDVI}=\rho_{i}-\rho_{r}\cdot \frac{\rho_{si}}{\rho_{sr}}$		[22]
Leaf Area Index	$\text{LAI}=-\frac{1}{\alpha^{*}}ln\left(1-\frac{\text{WDVI}}{\text{WDV}I_{\infty }}\right)$	α*, WDVI∞	[22]
Soil Adjusted vegetation index	SAVI = (ρi − ρr)/(ρi + ρr + 0.5)		[23]
Spectrally integrated hemispherical reflectance (*albedo*)	r = ∑λ wλ · ρλ	wλ	[24]

* *ρ_r_, ρ_i_ ρ_sr_, ρ_si_* represent reflectance in the red and infrared region for vegetation and soil respectively; α* is the extinction coefficient; *w_λ_* are weighted percentages of the extraterrestrial solar irradiance *E^0^_λ_* in each band of the sensor.

**Table 2.** Coordinates for the four corners of the Landsat images located in UTM zone 33N.

**Corner coordinates (WGS 84)**	**X**	**Y**
Upper left	472006	4155746
Upper right	508658	4155746
Lower left	472006	4127050
Lower right	508658	4127050

**Table 3.** TM Landsat-5 TM postcalibration features.

**Sensor**	**Pixel size (m)**	**Band**	**Band range (μm)**	Gain*(W m^-2^ sr^-1^ μm^-1^)	***Offset* (W m^-2^ sr^-1^ μm^-1^)**
Landsat 5 TM	30	1	0.45-0.52	0.6023	-1.50
		2	0.52-0.60	1.1749	-2.80
		3	0.63-0.69	0.8058	-1.20
		4	0.76-0.90	0.8145	-1.50
		5	1.55-1.75	0.1087	-0.37
	120	6	10.4-12.5	0.0551	1.20
	30	7	2.08-2.35	0.0569	-0.15

* Gain and Offset are band-specific rescaling factors typically given in the product header file

**Table 4.** Mean values of satellite-based vegetation indicators and the field measurements of LAI.

**Satellite-based indicators**	**Mean values**							

	June 14^th^		July 22^nd^		August 17^th^		September 8^th^	

	M	C_V_	M	C_V_	M	C_V_	M	C_V_

*albedo* (α)	0.18	0.08	0.16	0.05	0.17	0.04	0.12	0.07
*emissivity* (ε)	0.96	0.05	0.96	0.03	0.96	0.03	0.97	0.05
Leaf area index (LAI)	1.68	0.17	1.79	0.21	1.55	0.15	1.38	0.15
NDVI	0.50	0.07	0.55	0.09	0.60	0.08	0.45	0.06
SAVI	0.23	0.06	0.23	0.08	0.23	0.05	0.19	0.07
WDVI	0.17	0.11	0.20	0.07	0.24	0.14	0.17	0.10
Field measurements of LAI	1.45	0.16	1.40	0.15	1.55	0.17	1.35	0.18

* M: mean value;

** C_V_: coefficient of spatial variation from the mean value
